# Optimization and Performance Assessment of a Low-Dose Alanine Electron Paramagnetic Resonance Dosimetry Procedure for Potential Application in Electron Beam FLASH Radiotherapy

**DOI:** 10.3390/molecules31214029

**Published:** 2026-11-09

**Authors:** Babedi Sebinanyane, Urszula Gryczka, Chamunorwa Oscar Kureba, Gregory Campbell Hillhouse, Aneta Sterniczuk, Marta Walo

**Affiliations:** 1 Department of Physics and Astronomy, School of Pure and Applied Sciences, Botswana International University of Science and Technology, Plot 10071, Boseja Ward, Private Bag 16, Palapye, Botswana; 2 Institute of Nuclear Chemistry and Technology, ul. Dorodna 16, 03-195 Warszawa, Poland

**Keywords:** alanine, Electron Paramagnetic Resonance (EPR), low-dose, dosimetry, Ultra-High dose rate (UHDR), FLASH radiotherapy

## Abstract

Alanine Electron Paramagnetic Resonance (EPR) is an established reference and transfer dosimetry method in high-dose applications, valued for its high accuracy and long-term stability. However, it exhibits increased uncertainties for doses below 10 Gy, where the Signal-to-Noise Ratio (SNR) is reduced. This work presents the optimization and performance assessment of an alanine EPR measurement procedure for quantitative dose measurements below 10 Gy using a traceable ^60^Co reference irradiation source under conventional irradiation conditions. The effects of pellet position within the resonant cavity, number of pellets measured simultaneously, microwave power, modulation amplitude, and acquisition time were systematically investigated. The optimized procedure used a two-pellet configuration positioned at a cavity holder control screw length of 9 mm, a microwave power of 1.8 mW, and a modulation amplitude of 0.4 mT. The total spectral acquisition time was 20 min, made of 120 s sweep time and 10 accumulated scans for a magnetic field sweep width of 2 mT. Although consistent spectral resolution was achieved at 1 Gy, exploratory measurements from 1 to 10 Gy showed an uncertainty of 15.88% (*k* = 2), providing a coverage probability of approximately 95%. This did not support the establishment of a reliable quantitative calibration. As such, a practical calibration range of 4–10 Gy was then established using dose points at 4, 5, 6, 8, and 10 Gy and verified at 4.5, 7.0, and 9.0 Gy, with absolute percentage dose differences ranging from 0.00 to 4.44%. A repeatability test assessed at 7 Gy gave a relative standard deviation of 0.77%, indicating consistent EPR response under repeated measurements for the optimized procedure. The uncertainty of measurements in the range of 4–10 Gy was determined to be 5.40%, with a coverage factor of *k* = 2. These findings demonstrate the performance of the optimized low-dose alanine EPR procedure over a practical range of 4–10 Gy. This supports further investigation of alanine EPR as a potential independent dosimetry method for electron beam FLASH RT studies.

## 1. Introduction

Irradiation of alanine produces stable free radicals with concentrations proportional to the absorbed dose. These radiation-induced radicals contain unpaired electrons that can be quantified through Electron Paramagnetic Resonance (EPR) spectroscopy, producing a signal with an intensity proportional to the radical concentration [1]. Alanine EPR has been a reliable dosimetry method to quantify absorbed dose in several National Metrology Institutes (NMIs) [2,3]. Its desirable dosimetric properties include the following: (i) stability of radiation-induced radicals, (ii) a linear dose response for doses up to 10^5^ Gy, (iii) minimal energy dependence for absorbed dose to water measurements, and (iv) the use of the non-destructive readout method [4,5]. Furthermore, similarity of the radiation response between alanine and the biological tissue makes alanine EPR dosimetry attractive for radiotherapy (RT) applications. Clinical studies have demonstrated the feasibility of alanine EPR dosimetry for quality assurance in proton therapy [6] and in vivo photon and electron dose verification [7]. The minimal dose-rate dependence of alanine has positioned it as a potential passive dosimeter under FLASH RT, where radiation energy is delivered at ultra-high dose rates (UHDRs), typically ≥ 40 Gy/s [8]. Alanine EPR has subsequently been investigated for dosimetry in FLASH RT [9,10,11,12].

FLASH RT has gained considerable interest due to its ability to reduce normal tissue toxicity while maintaining tumor control. This has been demonstrated in different species such as the first human patient [13], mice [14], zebrafish [15] and mini pigs and cats [16]. In comparison to conventional RT, which commonly delivers radiation energy in daily fractions of approximately 1–2 Gy over several days or weeks, FLASH RT delivers radiation energy within very short irradiation times, reducing the total treatment time [15,17]. However, one of the major challenges limiting the standardization of FLASH RT is accurate dosimetry. Ionization chambers, which are widely used as reference dosimeters and real-time monitoring systems in conventional RT, suffer from ion recombination and saturation effects at UHDR [10,18]. As such, conventional ionization chambers provide inaccurate dose determination under FLASH RT conditions. Several laboratories have developed or modified real-time beam monitoring systems to operate under FLASH RT conditions [19,20,21]. Even so, standardized dosimetry protocols for these systems under FLASH RT conditions remain under development. Independent dosimetry systems are therefore essential to validate real-time beam-monitoring systems, support beam commissioning, and verify doses delivered under UHDR irradiation conditions. Although many FLASH RT studies involve doses above 10 Gy, accurate measurements below 10 Gy remain important for characterizing the lower part of the therapeutic dose range, investigating FLASH effect dose thresholds and supporting future treatment strategies involving lower fractional doses.

A major limitation of alanine EPR at low doses is the reduction in signal intensity caused by the lower concentration of radiation-induced radicals, resulting in a reduced Signal-to-Noise Ratio (SNR) and increased measurement variability [22]. Previous studies have shown that optimization of EPR acquisition parameters can improve alanine EPR performance [10,23]. Gondré et al. optimized alanine EPR measurements mainly over the 10–100 Gy range and reported reduced performance at 4.9 Gy [10], while Smith et al. demonstrated increased measurement variability as the dose decreased below 10 Gy [23]. These findings indicate that low-dose alanine EPR measurements remain challenging and that optimal acquisition settings are system dependent and may vary depending on the spectrometer, resonant cavity, dosimeter characteristics, and measurement configuration. Consequently, measurement parameters reported for one EPR system cannot be transferred directly to another system without experimental verification and adjustment.

This work aims to establish the practical quantitative dose range of an alanine EPR dosimetry system below 10 Gy through systematic optimization and performance evaluation under conventional irradiation conditions. Factors affecting measurement performance, including pellet positioning inside the EPR resonant cavity, total pellet mass evaluated by the number of pellets per measurement, microwave power, modulation amplitude, and measurement acquisition time were systematically optimized. The optimized procedure was subsequently evaluated through calibration, calibration verification, uncertainty assessment and repeatability measurements to determine its performance and practical lower limit for quantitative dose measurement.

## 2. Materials and Methods

The experimental procedure in this study included irradiation of alanine dosimeters and optimization of EPR parameters for low-dose measurements. The optimized procedure was subsequently used to establish and verify the calibration function. In addition, the associated measurement uncertainty and short-term repeatability were evaluated.

### 2.1. Dosimeter Preparation and Irradiation

Alanine pellets purchased from Aerial (Illkirch-Graffenstaden, France) were used for dosimetry measurements in this study. The pellets had an average mass of 37.70 ± 0.21 mg, diameter of 4.030 ± 0.004 mm, thickness of 2.389 ± 0.049 mm, and density of 1.247 ± 0.005 g/cm^3^. To maintain consistency throughout this study, all measurements were performed using pellets from the same production batch. For optimization and performance assessments, alanine pellets were irradiated using a ^60^Co reference source at conventional dose-rates ranging from 0.0261 to 0.0323 Gy/s over the experimental period. The ^60^Co source was selected as the traceable reference irradiation system available at the Laboratory for Measurements of Technological Doses (LMTD) of the Institute of Nuclear Chemistry and Technology (INCT) in Poland, where the response of alanine reference dosimeters is traceable to national standards at the National Physical Laboratory (NPL), United Kingdom. This provided well-characterized absorbed doses for systematic optimization and performance assessment of the low-dose alanine EPR procedure. Previous studies have reported small radiation quality effects for alanine between ^60^Co photons and high-energy electron beams, with relative response scaling factors closer to unity [24,25].

### 2.2. Alanine EPR Measurements

The dose in the irradiated alanine pellets was measured using a Magnettech Miniscope MS5000 X-band spectrometer equipped with a TE102 rectangular cavity [26]. The spectrometer was operated via ESRStudio software (version 1.81.3) for spectral visualization during optimization and the Aerial AerEDE software (version 5.3.4) for calibration and dose measurements. All EPR measurements were performed at a room temperature of about 22 °C, a relative humidity of 40%, and an EPR resonant cavity temperature of about 31 °C.

### 2.3. Optimization of Parameters for Measurement

#### 2.3.1. Pellet Position in the Resonant Cavity

**Figure 1:**
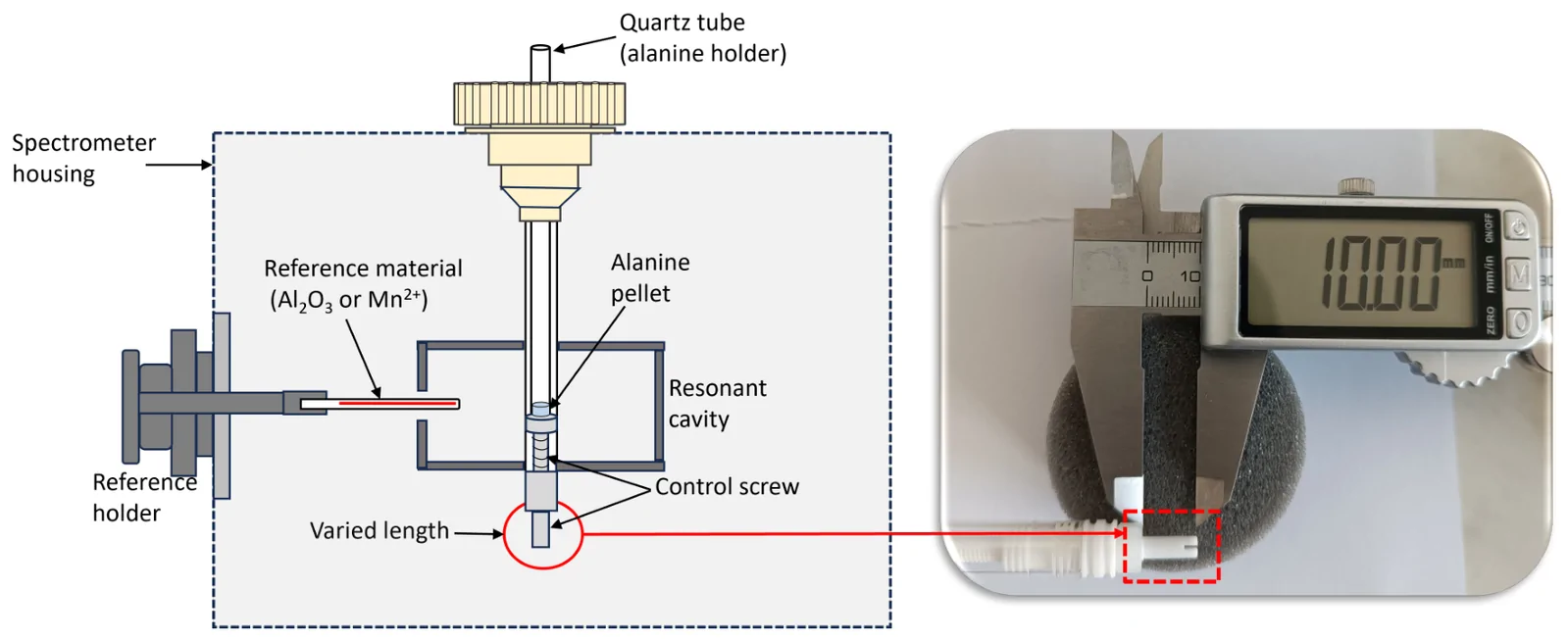
A schematic illustrating the alanine positioning procedure within the resonant cavity. Pellet position was varied by adjusting the cavity holder position control screw length, with each position defined by the measured screw length.

The position of a 20 Gy pre-irradiated alanine pellet of the same type and geometry as those used throughout this study was varied along the vertical axis of the EPR resonant cavity using the cavity holder position control screw, as illustrated in Figure 1. The purpose of this separate dose selection was to provide a strong and clearly detectable EPR signal across the full positional range of the resonant cavity, including positions with reduced sensitivity. Reported positions correspond to the measured length of the control screw. The observed positional trend was to identify a maximum response area. EPR operational parameters recommended from the manufacturer’s manual for a dose range of <1–100 Gy were fixed at a microwave power of 10 mW and a modulation amplitude of 0.7 mT [26]. Other parameters were fixed at a sweep time of 240 s, a single scan, and a magnetic field sweep width of 15 mT. The position was adjusted in 2 mm increments and reduced down to 1 mm towards the center of the cavity. At each position, three repeated measurements were acquired to assess the variability of the EPR signal’s intensity based on the height of the central alanine peak.

#### 2.3.2. Number of Pellets per Measurement

The effect of increasing the total pellet mass on the EPR signal intensity was evaluated by increasing number of simultaneously measured 10 Gy irradiated pellets. Intensity measurements were performed using different pellet configurations and parameters described in Section 2.3.1 above. For each pellet configuration, three repeated measurements were conducted to assess variability. Based on this optimization, all subsequent optimization and performance assessment in this study were performed using the optimized number of pellets.

#### 2.3.3. Microwave Power

A microwave power response was evaluated using 10 Gy irradiated pellets and the pellet configuration selected in Section 2.3.2. Microwave power values in the range of 0.1–10 mW were investigated for modulation amplitudes ranging from 0.1 to 1.0 mT. All measurements were acquired for a sweep time of 60 s, two averaged accumulations, and across a magnetic field sweep width of 15 mT, which recorded the full undistorted alanine spectra for quantification. At each combination of the power setting and modulation amplitude, the intensity of the signal was determined using the height of the central alanine peak.

#### 2.3.4. Modulation Amplitude

Using parameters optimized in Section 2.3.1, Section 2.3.2 and Section 2.3.3, 10 Gy irradiated pellets were evaluated for spectral broadening and signal enhancement across modulation amplitude ranging from 0.1 to 1 mT. All measurements were carried out for a sweep time of 60 s, two averaged accumulations, and across a fixed magnetic field sweep width of 2 mT. These parameters accommodated only the full central alanine peak, clearly showing the maximum and minimum of the peak for accurate signal intensity quantification. A suitable modulation amplitude was selected based on evaluation of the Full Width at Half Maximum (FWHM) and the EPR signal intensity trend against increasing modulation.

#### 2.3.5. Measurement Time

The total acquisition time for accurate detection of the peak-to-peak height of the central alanine peak was optimized at 1 Gy, where reliable peak detection is challenging due to reduced SNR. This was evaluated by varying different combinations of the sweep time and the number of accumulated scans. Five independent measurements for a field range of 2 mT were performed for a series of increasing acquisition times, starting from 60 s and 2 accumulations which was utilized in modulation amplitude optimization. Before each measurement, the quartz tube was removed and re-inserted into the EPR resonant cavity to account for repositioning effects. For each acquisition time, the resulting spectrum was recorded and visually assessed for clear resolution and for reproducibility of both the maximum and the minimum of the peak. The acquisition time was increased until a reproducible outcome was met across all five measurements.

### 2.4. Calibration

Calibration was initially performed over the dose range of 1–10 Gy using the Aerial AerEDE calibration and dose evaluation software (version 5.1.0) [27]. The calibration function described the relationship between the nominal absorbed dose and the EPR response. The EPR response was defined as the alanine signal corrected using the internal ruby reference to account for variations in spectrometer sensitivity and subsequently normalized to the total pellet mass. Calibration dose point selection was guided by the ASTM 51261 [28], which recommends that the minimum number of dose points, *N*, should satisfy the following:

(1) N=5×Q,

where

(2) $$Q=\log_{10}\left(\frac{D_{\max}}{D_{\min}}\right),$$

with $D_{\max}$ and $D_{\min}$ being the maximum and minimum doses in the calibration range. If Q≥ 1, *N* is rounded up to the nearest integer, and if Q< 1, *N* should be five points. In this study, $D_{\max}=10$ Gy and $D_{\min}=1$ Gy, giving Q=1. Eight calibration points were therefore selected to fully cover the intended calibration range with smaller dose intervals at the lower-dose region, where more noticeable signal variability was expected. The selected doses were 1, 2, 3, 4, 5, 6, 8, and 10 Gy. Calibration was performed using four independent dosimeters at each dose point.

The calibration function was obtained by linear regression of the mass normalized EPR response against the nominal absorbed dose. The precision of the fitted calibration coefficients, that is, the slope and the intercept, was evaluated using their 95% confidence intervals. For each coefficient $C_{i}$, the confidence interval was calculated as $C_{i}\pm t_{0.975,n-2}\times SE\left(C_{i}\right)$, where $SE(C_{i})$ is the standard error of the fitted coefficient, *n* is the number of individual calibration measurements, and $t_{0.975,n-2}$ is the two-sided Student-t critical value for a 95% confidence level.

Percentage response residual plot was created to assess the adequacy of the fitted calibration function and to identify dose dependent deviations. The individual points were calculated using the equation:

(3) $$r_{i}\left(\%\right)=\frac{y_{i}-{\hat{y}}_{i}}{{\hat{y}}_{i}}\times 100,$$

where $r_{i}$ is the response residual, $y_{i}$ is the measured response, and ${\hat{y}}_{i}$ is the response predicted by the calibration function.

Due to greater uncertainties in the range of 1–10 Gy, a practical calibration range was later established for the dose range of 4–10 Gy with five calibration points of 4, 5, 6, 8 and 10 Gy. This satisfied the minimum dose-point recommendation of ASTM 51261. However, increasing the number of independent dosimeters at these dose levels would provide a more robust estimate of the calibration fit and dosimeter-to-dosimeter variability. A percentage response residual plot was also created for this calibration range.

#### 2.4.1. Uncertainty Assessment

The measurement uncertainty of the LMTD alanine EPR dosimetry system in dose ranges 1–10 Gy and 4–10 Gy were assessed based on (i) calibration of doses from the laboratory certificate ($u_{\mathrm{cert}}$), (ii) fitting of the calibration function ($u_{\mathrm{fit}}$), (iii) dosimeter-to-dosimeter scatter (reproducibility) ($u_{\mathrm{scatter}}$), (iv) pellet mass measurement ($u_{\mathrm{mass}}$), and (v) the signal correction with the ruby reference material ($u_{\mathrm{ruby}}$). The calibration dose uncertainty represented the combined uncertainty associated with the reference irradiation and absorbed dose delivery system assigned by the calibration laboratory. The components $u_{\mathrm{fit}}$, $u_{\mathrm{scatter}}$, and $u_{\mathrm{ruby}}$ were obtained from the calibration software, while $u_{\mathrm{cert}}$ and $u_{\mathrm{mass}}$ were obtained from laboratory calibration certificates. Each component was expressed as an uncertainty with a coverage factor, k=1. The uncertainties were expressed as Type A or Type B, where Type A was determined from calibration measurements and Type B was obtained from existing information from calibration certificates or software. The combined uncertainty, $u_{c}$, was then calculated using the following equation:

(4) $$u_{c}=\sqrt{u_{\mathrm{cert}}^{2}+u_{\mathrm{fit}}^{2}+u_{\mathrm{scatter}}^{2}+u_{\mathrm{mass}}^{2}+u_{\mathrm{ruby}}^{2}}$$

The expanded uncertainty, *U*, was obtained using

(5) $$U=ku_{c}$$

where a coverage factor of k=2 was applied, providing a confidence interval of approximately 95%.

#### 2.4.2. Calibration Verification

To verify the calibration function, alanine pellets independently irradiated at 4.5, 7.0, and 9.0 Gy were used. These doses were selected as intermediate points to assess the ability of the calibration function to estimate the absorbed dose at the lower, middle, and upper regions of the investigated range. Four dosimeters were measured at each verification dose using the optimized measurement procedure. One dosimeter was made of a pellet configuration selected in Section 2.3.2. The calibration line was fitted linearly, and the absorbed dose for the verification points was determined in the software by the following equation:

(6) $$D=\frac{r-c}{m},$$

where *D* is the calculated dose in Gy; *r* represents the corrected, mass normalized EPR response; *c* represents the y-intercept; and *m* represents the slope.

#### 2.4.3. Repeatability Assessment

Using the optimized procedure, measurement repeatability was assessed at 7 Gy, a midpoint of the 4–10 Gy calibration range. This evaluation was performed to determine the short-term precision of the optimized procedure under identical experimental conditions. The same pellet configuration was measured ten times, with removal and reinsertion of the quartz tube (measurement tube) into the resonant cavity before each acquisition. This was carried out to include variability associated with sample repositioning. Repeatability of measurements was evaluated from the mean, standard deviation, and Relative Standard Deviation (RSD) of the ruby-corrected, mass normalized EPR response.

## 3. Results and Discussion

In this study, factors affecting the performance of alanine EPR dosimetry at low doses were optimized to improve measurement reliability below 10 Gy. The optimized procedure was used to establish a practical quantitative measurement range under conventional ^60^Co irradiation conditions. Following optimization of the measurement conditions, all subsequent measurements were carried out using the selected parameters. The resulting procedure may subsequently be evaluated under UHDR conditions for potential application in electron beam FLASH RT dosimetry.

### 3.1. Optimization of Parameters for Measurement

#### 3.1.1. Pellet Position Inside EPR Resonant Cavity

**Figure 2:**
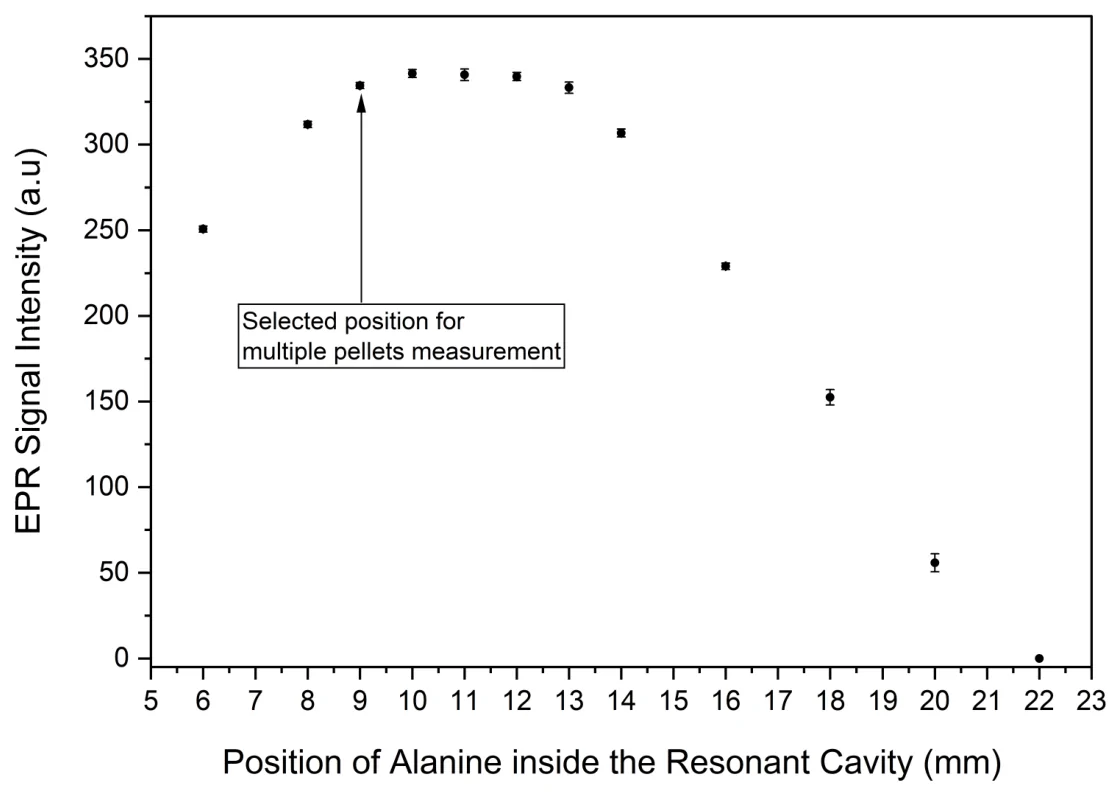
The effect of pellet position on the EPR signal intensity. Error bars represent a standard deviation of three measurements.

The effect of the alanine pellet position within the EPR resonant cavity on the intensity of the signal is shown in Figure 2. The intensity of the signal increased with increasing control screw length, reaching a high response region from 9 to 13 mm and subsequently declined. Comparable signal intensities observed over the 9–13 mm range indicate a region of high cavity sensitivity. A position of 9 mm was therefore selected as an operational position since it marked the beginning of the high-response region, allowing sufficient space for evaluation of multiple stacked pellets to increase detectable signal at low doses.

#### 3.1.2. Number of Pellets per Measurement

**Figure 3:**
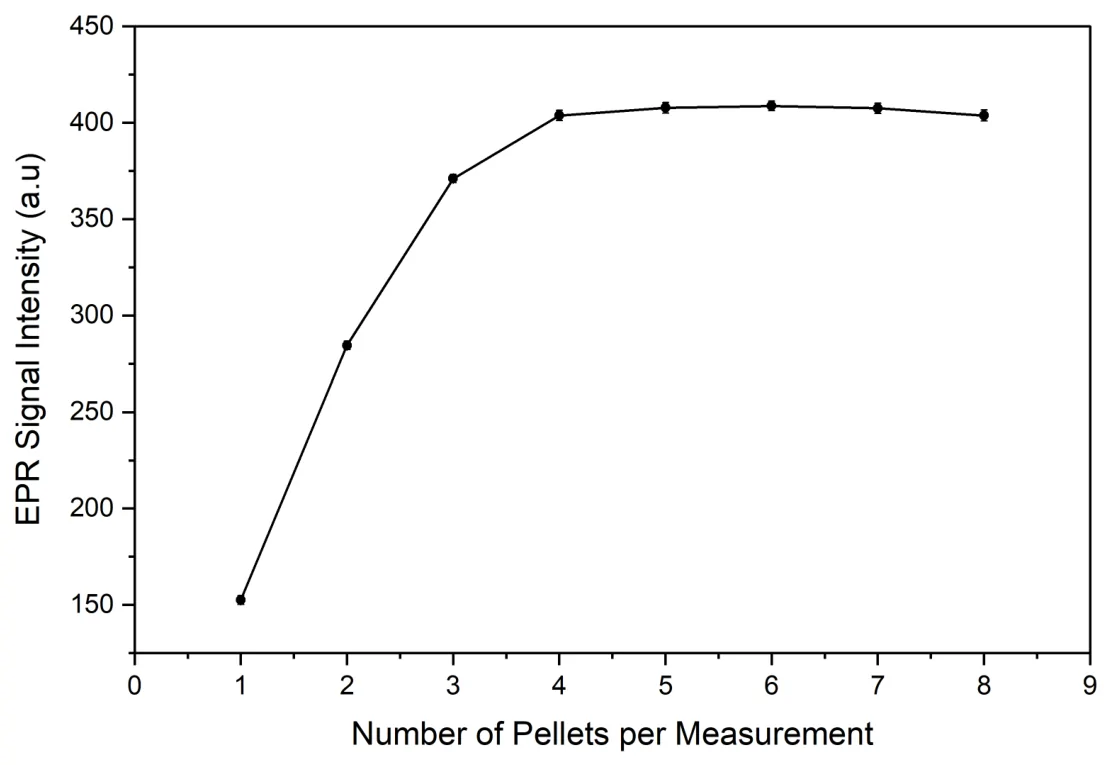
The effect of increasing number of pellets per measurement on the EPR signal intensity for 10 Gy irradiated pellets. The total pellet mass also increases with the number of pellets per measurement. Error bars represent a standard deviation of three measurements.

Figure 3 shows the effect of increasing total pellet mass, determined by the number of pellets per measurement on the EPR signal intensity. As the number of pellets per measurement increases, the total radical concentration inside the cavity also increases, resulting in an increase in the EPR signal’s intensity. In theory, the EPR signal intensity should increase linearly with an increase in the number of pellets measured due to similar mass concentrations in each pellet. However, the results show that the EPR signal’s intensity significantly increases up to four stacked pellets, and a further increase in number of pellets provides minimal additive contribution of signal intensity. This arises from overfilling of the cavity, where the stacked pellets extend beyond the sensitive region of the resonant cavity. Although a four-pellet configuration produced a higher signal intensity, the incremental signal gain decreased as additional pellets were added, indicating that the stacked sample increasingly extended beyond the most sensitive region of the cavity. A two-pellet configuration was selected as it provided a substantial signal increase while maintaining the closest proportional relationship between signal intensity and total pellet mass. This configuration also limits the loss of incremental response associated with cavity overfilling and stacked pellet placement relative to the sensitive region.

#### 3.1.3. Microwave Power

**Figure 4:**
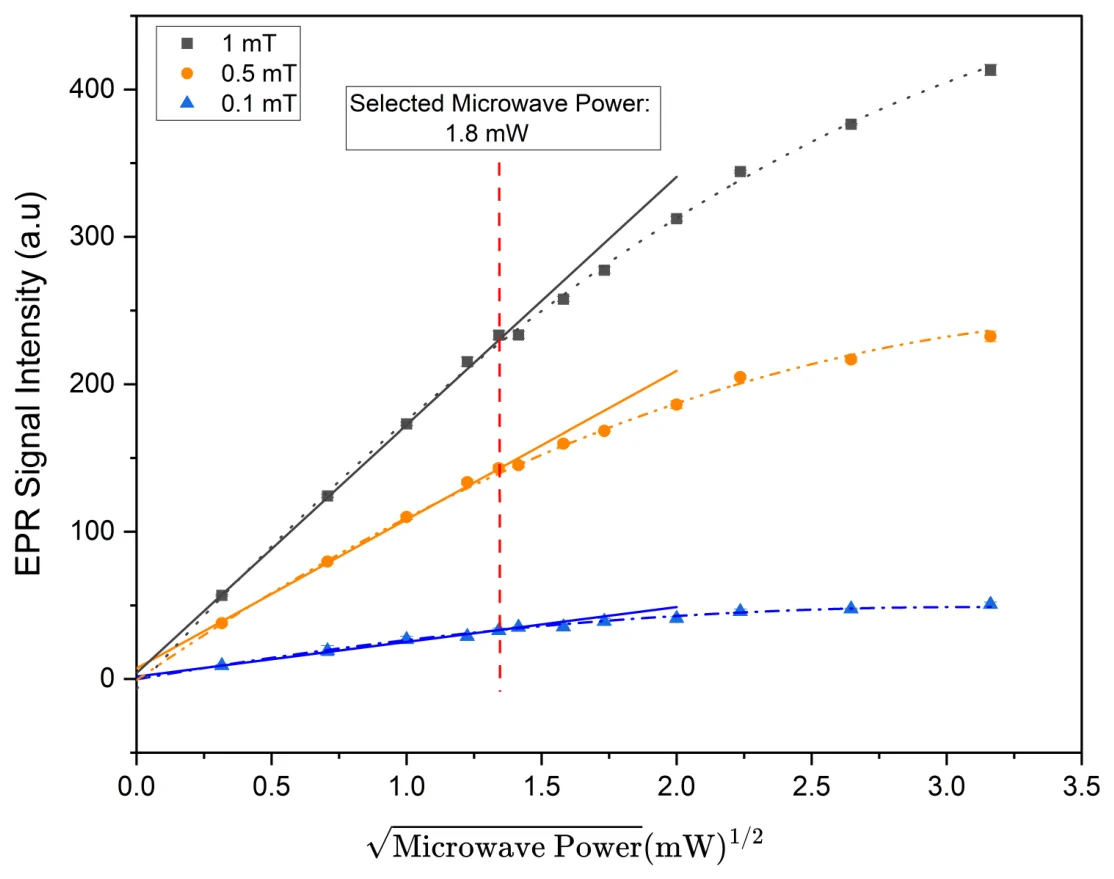
EPR signal intensity of the central alanine peak as a function of the square root of microwave power at three representative modulation amplitudes selected from the investigated range of 0.1–1.0 mT. The polynomial curves illustrate the experimental response, solid straight lines represent extrapolated linear fits of the first six power points and the dashed vertical line indicates the selected microwave power of 1.8 mW.

A microwave power saturation trend was evaluated using the EPR signal intensity of the central alanine peak as a function of the square root of microwave power for varying modulation. Three representative modulation amplitudes were selected and plotted in Figure 4 to assess the power saturation trend on the investigated range of 0.1–1.0 mT. The polynomial curves at each modulation amplitude represent the overall experimental response, while the solid lines indicate linear fits to the first six low power levels, representing the non-saturated response region. These linear fits were extrapolated to illustrate deviations from linearity at higher powers. A microwave power setting of 1.8 mW was selected as the highest investigated operating value that remained within the approximately linear low-power response region, thereby providing signal enhancement while limiting microwave saturation effects.

#### 3.1.4. Modulation Amplitude

**Figure 5:**
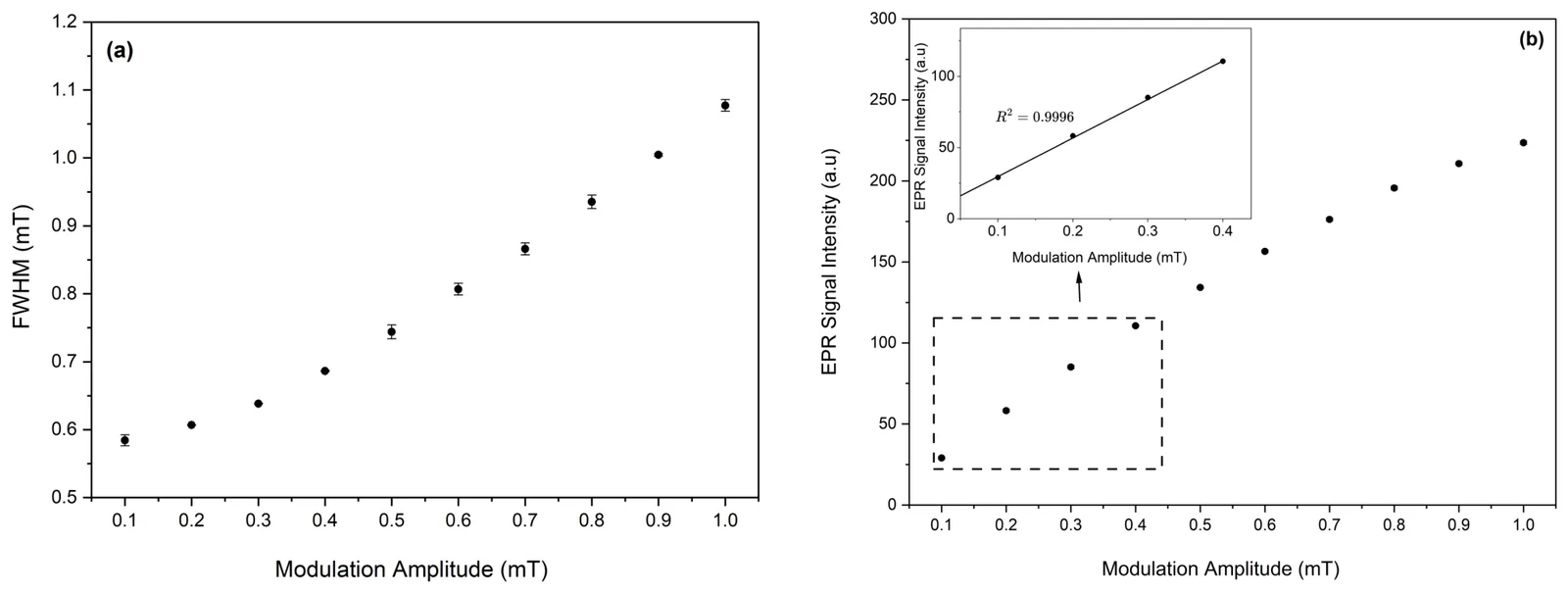
The effect of modulation amplitude on the (**a**) Full Width at Half Maximum (FWHM) and (**b**) EPR signal intensity. Error bars represent a standard deviation of three measurements.

The effect of increasing modulation amplitude on the peak width (determined by the FWHM) and the intensity of the central alanine peak are shown in Figure 5a,b, respectively. The FWHM increased gradually from 0.58 mT at a modulation amplitude of 0.1 mT to 1.08 mT at 1.0 mT, indicating progressive modulation-induced broadening of the EPR spectrum. The experimental results do not show a well-defined threshold marking the onset of overmodulation within the investigated range. Thus, evaluation of the FWHM alone did not provide a clear basis for selecting a practical modulation amplitude. An increase in modulation amplitude also increased the intensity of the signal, improving the SNR. In the range of 0.1–0.4 mT, the EPR signal intensity increased linearly with a coefficient of determination $R^{2}=0.9996$, indicating efficient signal enhancement. Above 0.4 mT, the signal intensity continued to increase, but the incremental signal gain progressively decreased, while the linewidth continued to broaden. A modulation amplitude of 0.4 mT was therefore selected as the highest value within the initial linear signal enhancement region. This modulation setting provided substantial signal enhancement while minimizing modulation induced broadening to preserve the central alanine line shape for reproducible determination of the peak intensity.

#### 3.1.5. Measurement Time at 1 Gy

**Figure 6:**
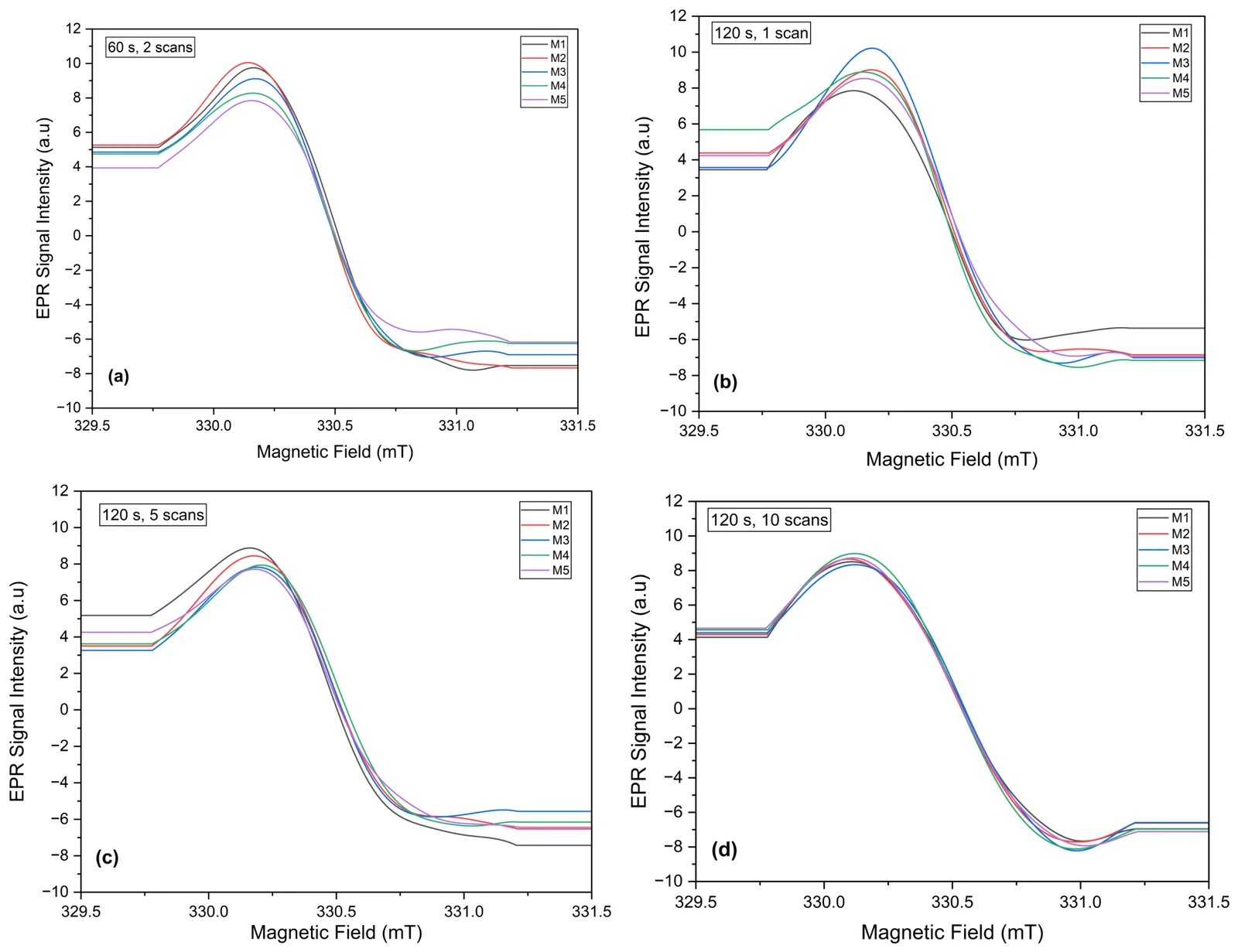
EPR spectra of alanine pellets irradiated at 1 Gy for increasing measurement times: (**a**) 60 s and 2 scans, (**b**) spectra acquired for increased sweep time 120 s and 1 scan, (**c**) 120 s and 5 scans, and (**d**) the selected 120 s and 10 scans. M1–M5 represent five independent measurements with removal and re-insertion of the measurement tube between measurements.

The effect of increasing the total acquisition time on the spectral shape of two stacked 1 Gy irradiated pellets is shown in Figure 6a–d. Measurement time of 60 s and two accumulated scans utilized in modulation amplitude optimization was first evaluated and resulted in insufficient signal definition. To improve signal resolution, the sweep time was increased to 120 s, and the number of scans was subsequently varied. At 120 s with single and five scans, the peak remained poorly resolved. At 10 scans, the spectral shape showed a consistent resolution with clearly defined minima and maxima across all measurements. This minimized the likelihood of the software selecting distorted or noise induced points when determining the peak-to-peak intensity. Thus, an acquisition condition of 120 s and 10 scans, corresponding to 20 min, for a magnetic field sweep width of 2 mT was selected as the shortest condition to provide a reproducible resolution of the central alanine peak for accurate dose determination. With increasing dose, the total measurement acquisition time is expected to reduce due to improved SNR, leading to faster measurement times.

### 3.2. Calibration

**Figure 7:**
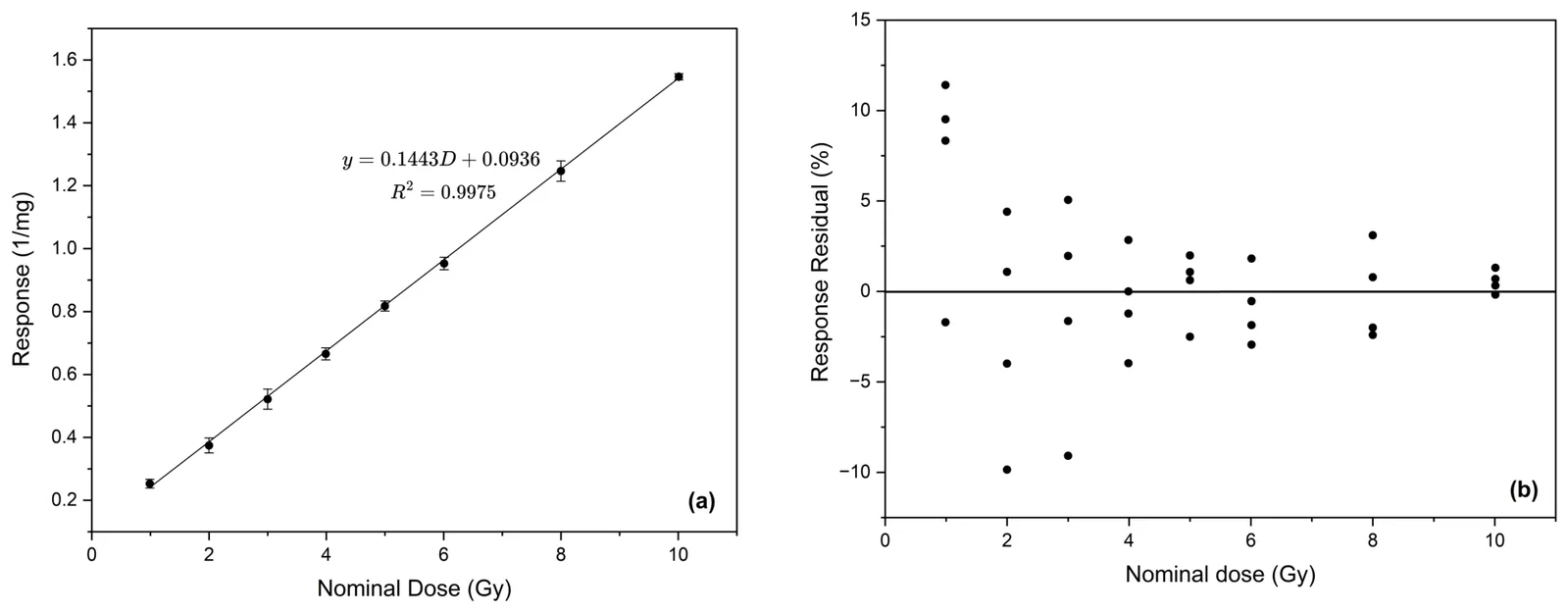
Calibration and the corresponding response residual plot (**a**) mass normalized EPR response curve against nominal absorbed dose over the calibration range of 1–10 Gy. The data points represent the mean response at each dose, and the error bars indicate the standard deviation of the individual responses. (**b**) Percentage residuals of the individual responses relative to the fitted calibration function.

Before establishing the final calibration range, exploratory measurements for the dose range 1–10 Gy were performed using the optimized procedure. A calibration curve was generated by plotting the ruby-corrected and normalized mass response of the alanine pellets as a function of the nominal absorbed dose, as illustrated in Figure 7a. The pellet response exhibited a linear regression with dose and was described by the equation y=0.1443D+0.0936, with a coefficient of determination of $R^{2}=0.9975$, where *y* is the normalized EPR response and *D* is the absorbed dose in Gray (Gy). The fitted slope was 0.1443, with a 95% confidence interval from 0.1416 to 0.1470, while the intercept was 0.0936, with a 95% confidence interval from 0.0783 to 0.1090. The coefficient confidence intervals show that the slope was estimated more precisely than the intercept. Despite a good calibration fit for the range 1–10 Gy with the optimized procedure, the percentage residuals shown in Figure 7b indicate measurement variability of approximately up to 10% for 1–3 Gy. This leads to an increase in the overall uncertainty for the calibration discussed further in Section 3.2.1 and hindered reliable calibration for the optimized procedure in the range of 1–10 Gy. However, the percentage response residuals for individual measurements reduced down to <5% for doses beyond 3 Gy.

**Figure 8:**
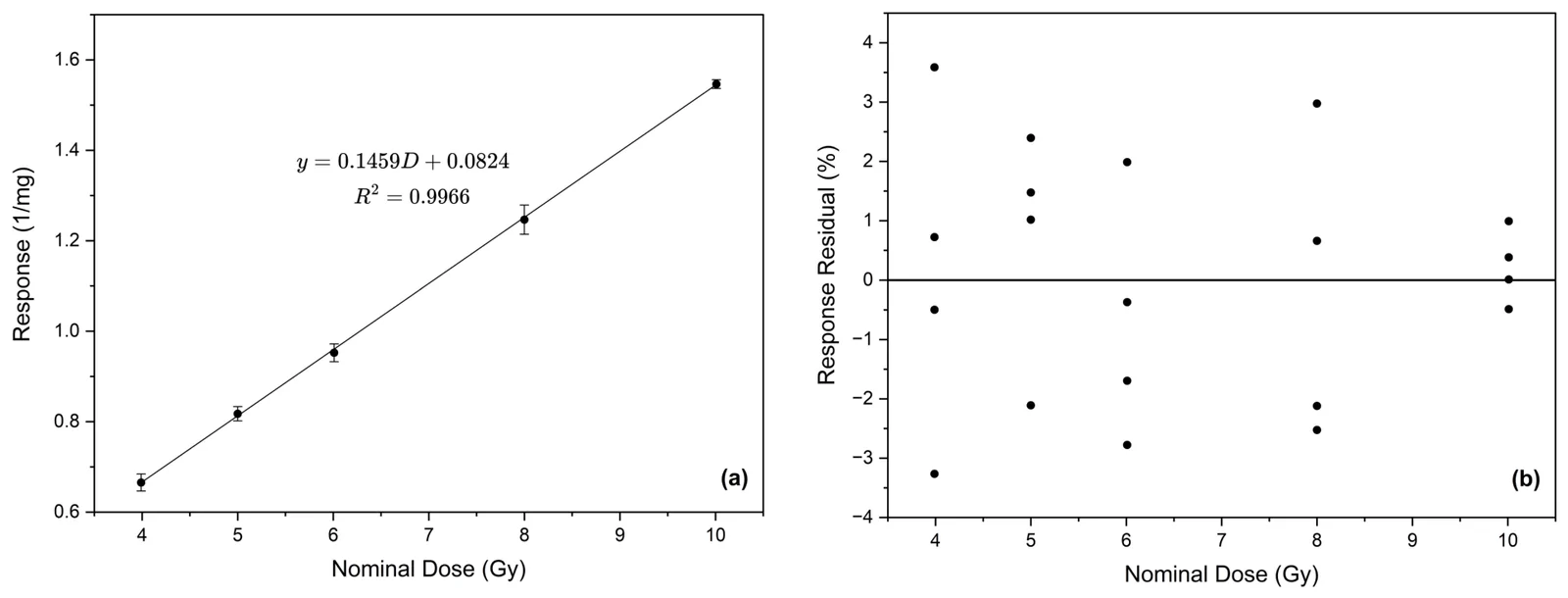
Calibration and the corresponding response residual plot (**a**) calibration fit for the dose range 4–10 Gy using optimized parameters. Error bars indicate the standard deviation of the individual responses. (**b**) Percentage residuals of the individual responses relative to the fitted calibration function.

Another calibration curve was created for a dose range of 4–10 Gy, as shown in Figure 8a. The response of the measured pellet configuration showed a linear regression with the nominal dose and was described by the equation y=0.1459D+0.0824, with a coefficient of determination of $R^{2}=0.9966$. For this fit, the 95% confidence interval ranged from 0.1417 to 0.1501 for the slope and from 0.0531 to 0.1118 for the intercept. These confidence intervals indicate that the slope was estimated with higher precision than the intercept. The high coefficient of determination indicates that the linear regression accounted for most of the variation in the measured response within the investigated range. Percentage response residuals of calibration measurements for the range 4–10 Gy are shown in Figure 8b. The residuals were distributed on both sides of the zero throughout the calibration range, indicating no consistent monotonic trend of the alanine response. A relatively larger spread was observed between 4 and 8 Gy, while the responses were closely distributed around the fitted calibration line at 10 Gy. This is consistent with greater measurement variability and reduced SNR at doses less than 10 Gy. All response residuals remained within ±4%, with no outliers inconsistent with the overall distribution, indicating that the measured responses agreed closely with the fitted linear calibration function over the investigated range.

The five calibration dose points used over the 4–10 Gy range satisfy the minimum dose point recommendation of ASTM 51261. However, the relatively larger response scatter observed between 4 and 8 Gy indicates that greater replication would be particularly valuable near the lower boundary of the calibration range. Increasing the number of independently irradiated dosimeters at these dose levels would provide a more robust estimate of the calibration fit and dosimeter-to-dosimeter variability.

#### 3.2.1. Uncertainty Assessment

The uncertainty budget aimed at determining the reliability of the calibration functions is summarized in Table 1. The budget for the exploratory dose range of 1–10 Gy gives a combined standard uncertainty of 7.94% (k=1), resulting in an expanded uncertainty of 15.88% (k=2). This indicates that the dose determined using the measurement procedure should be reported with an uncertainty interval corresponding to 15.88% of the measured value. In conventional radiotherapy, an expanded uncertainty of approximately ±5% at a 95% confidence level is commonly cited as a benchmark for overall dose delivery [29]. The uncertainty estimated in the present study includes contributions from the reference irradiation, calibration, and alanine EPR measurement procedure. The commonly cited 5% value is therefore used as a clinical reference for interpreting the magnitude of the combined uncertainty. The relatively larger uncertainty indicates that the optimized procedure cannot produce a reliable calibration accuracy for the dose range of 1–10 Gy. The highest contributors to the calibration uncertainty come from the dosimeter-to-dosimeter scatter and the calibration fit, which is caused by the high variability of measurements in the lower range.

Table 1 further shows the uncertainty budget of the practical calibration range of 4–10 Gy. The expanded uncertainty was 5.40% (k=2), showing a considerable improvement compared with the exploratory 1–10 Gy range. Dosimeter-to-dosimeter scatter contributed 2.24% (k=1) to the overall uncertainty and remained the largest uncertainty component. This variability may reflect pellet-to-pellet differences, low SNR at the lower end of the calibration range, sample positioning within the resonant cavity, and short-term measurement variations. Spectrometer sensitivity variations were corrected using the internal ruby reference, with the corresponding uncertainty contribution included in the uncertainty budget.

#### 3.2.2. Calibration Verification

Table 2 presents the calibration verification results obtained using independently irradiated dosimeters at 4.5, 7.0, and 9.0 Gy. At 4.5 Gy, the measured doses ranged from 4.30 to 4.60 Gy, corresponding to absolute percentage deviations of 0.00% to 4.44%. At 7.0 Gy, the measured doses ranged from 6.90 to 7.20 Gy, with absolute percentage deviations of 1.43% to 2.86%. The measurements at 9.0 Gy ranged from 8.80 to 8.90 Gy, corresponding to absolute percentage deviations of 1.11% to 2.22%. The verification results demonstrate that the calibration function can estimate delivered nominal doses throughout the investigated range. The largest deviation was observed at 4.5 Gy, near the lower end of the calibration range, where the alanine response is weaker and consequently more sensitive to measurement variability. Agreement improved at 7.0 Gy, where the measured values were distributed on both sides of the nominal dose. At 9.0 Gy, all four measurements slightly underestimated the nominal dose, indicating a small systematic shift for this verification point. However, the magnitude of this underestimation remained within the expanded measurement uncertainty of 5.40%, which indicates consistency of the verification result with the calibration function. Overall, the results support the applicability of the calibration over the selected range of 4–10 Gy while also showing the expected increase in percentage deviation toward its lower limit. Although verification dose levels were distributed across the 4–10 Gy calibration range, additional calibration verification points, particularly near 4 Gy, would provide a more comprehensive assessment of calibration performance.

#### 3.2.3. Repeatability Assessment

Repeatability of measurements using the optimized procedure was evaluated for ten repeated measurements at a mid-dose of 7 Gy, with removal and re-insertion of the quartz tube between acquisitions. As shown in Table 3, the corrected mass normalized EPR response ranged from 1.1299 to 1.1584 mg^−1^, with a mean response of 1.1477 mg^−1^ and a standard deviation of 0.0088 mg^−1^. The corresponding RSD was 0.77%, indicating low variability and good short-term precision among repeated measurements performed under the same experimental conditions. This good precision may be attributed to a better SNR due to the use of the two-pellet configuration, positioning of the two pellets in a high-response region of the resonant cavity and signal averaging over a 20 min measurement time. These settings have collectively led to a reduced influence of sample repositioning on the EPR response, demonstrating a stable response determination at 7 Gy.

## 4. Conclusions

An alanine EPR measurement procedure was optimized and assessed for quantitative dose measurements below 10 Gy using a traceable ^60^Co reference irradiation source under conventional dose-rate conditions at the LMTD. The optimized procedure employed a two-pellet configuration positioned at a control screw length of 9 mm, with a microwave power of 1.8 mW and a modulation amplitude of 0.4 mT. The total acquisition time was 20 min, comprising a 120 s sweep time and 10 accumulated scans over a magnetic field width of 2 mT. Although the optimized parameters allowed consistent resolution of the central alanine peak at 1 Gy, the large response variability from 1 to 3 Gy resulted in an expanded uncertainty of 15.88% for the 1–10 Gy calibration range. Thus, measurements in this lower dose region did not support reliable quantitative calibration under the optimized procedure.

A practical quantitative calibration range was established for the dose range 4–10 Gy, with individual response residuals within ±4%. Calibration verification at 4.5, 7.0, and 9.0 Gy using independently irradiated dosimeters produced absolute percentage deviations ranging from 0.00 to 4.44%, verifying the ability of the calibration function to determine unknown doses within the calibration uncertainty. Repeatability assessed at 7 Gy yielded an RSD of 0.77%, demonstrating stable response determination under repeated measurement and repositioning conditions. The expanded measurement uncertainty was 5.40% at a coverage factor of k=2, representing a substantial improvement compared with the 15.88% uncertainty obtained over the 1–10 Gy range. This value is close to but slightly exceeds the commonly cited 5% uncertainty benchmark for overall dose delivery in conventional radiotherapy. Nevertheless, the calibration residuals, verification results, repeatability measurements, and substantial reduction in measurement uncertainty support the use of 4–10 Gy as the practical quantitative calibration range under the investigated conditions, with 4 Gy representing the lower practical limit. Further work should focus on minimizing measurement variability and its associated uncertainty, particularly at the lower end of the calibration range. The optimized procedure should also be evaluated under electron beam UHDR conditions to assess its potential application in FLASH RT dosimetry.

## Figures and Tables

**Table 1:** Uncertainty assessment for alanine EPR dose measurement over the 1–10 Gy and 4–10 Gy dose ranges.

Source of Uncertainty	Type	Uncertainty (%)	
		1–10 Gy	4–10 Gy
Calibration doses from laboratory certificate, $u_{\mathrm{cert}}$	B	1.30	1.30
Fit of calibration function, $u_{\mathrm{fit}}$	A	4.20	0.52
Dosimeter-to-dosimeter scatter, $u_{\mathrm{scatter}}$	A	6.59	2.24
Dosimeter mass measurement, $u_{\mathrm{mass}}$	B	0.28	0.28
Signal correction with ruby reference, $u_{\mathrm{ruby}}$	B	0.50	0.50
Combined uncertainty, $u_{c}$ (k=1)		7.94	2.70
Expanded uncertainty, *U* (k=2)		15.88	5.40

**Table 2:** Verification of the calibration curve using independently irradiated dosimeters at additional dose points.

Nominal Dose (Gy)	Dosimeter	Measured Dose (Gy)	Absolute Dose Difference (Gy)	Absolute Percentage Deviation (%)
4.5	A	4.40	0.10	2.22
	B	4.30	0.20	4.44
	C	4.60	0.10	2.22
	D	4.50	0.00	0.00
7.0	A	7.10	0.10	1.43
	B	7.20	0.20	2.86
	C	6.90	0.10	1.43
	D	7.10	0.10	1.43
9.0	A	8.90	0.10	1.11
	B	8.80	0.20	2.22
	C	8.80	0.20	2.22
	D	8.80	0.20	2.22

**Table 3:** Repeatability of the corrected mass normalized EPR response at 7 Gy.

Measurement Number	Mass Normalized Response (mg^−1^)
1	1.1299
2	1.1511
3	1.1380
4	1.1478
5	1.1481
6	1.1531
7	1.1584
8	1.1571
9	1.1511
10	1.1420
Mean	1.1477
Standard deviation	0.0088
RSD (%)	0.77

## Data Availability

Data is contained within the article.
